# Colorectal Cancer Liver Metastases: Is an R1 Hepatic Resection Accepted?

**DOI:** 10.3390/clinpract12060112

**Published:** 2022-12-19

**Authors:** Dimitrios Symeonidis, Konstantinos Tepetes, George Tzovaras, Labrini Kissa, Athina A. Samara, Effrosyni Bompou, Dimitrios Zacharoulis

**Affiliations:** Department of Surgery, University Hospital of Larissa, Mezourlo, 41110 Larissa, Greece

**Keywords:** colorectal cancer liver metastases, resection margins, R0 resection, R1 resection

## Abstract

Metastatic colorectal cancer is associated with a rather dismal 5-year overall survival. The liver is the most commonly affected organ. Improved 5-year survival rates after successful hepatic resections for metastases confined to the liver have been reported. Certainly, a hepatectomy that results in an incomplete tumor resection, in terms of leaving macroscopic residual tumor in the future liver remnant, is not associated with survival benefits. However, the prognostic implications of a microscopically positive surgical margin or a clear margin of less than 1 mm (R1) on pathology are debatable. Although it has been a field of extensive research, the relevant literature often reports contradictory results. The purpose of the present study was to define, assess the risk factors for, and, ultimately, analyze the effect that an R1 hepatic resection for colorectal cancer liver metastases might have on local recurrence rates and long-term prognosis by reviewing the relevant literature. Achieving an R0 hepatic resection, optimally with more than 1 mm of clear margin, should always be the goal. However, in the era of the aggressive multimodality treatment of liver metastatic colorectal cancer, an R1 resection might be the cost of increasing the pool of patients finally eligible for resection. The majority of literature reports have highlighted the detrimental effect of R1 resections on local recurrence and overall survival. However, there are indeed studies that degraded the prognostic handicap as a consequence of an R1 resection in selected patients and highlighted the presence of RAS mutations, the response to chemotherapy, and, in general, factors that reflect the biology of the disease as important, if not the determinant, prognostic factors. In these patients, the aggressive disease biology seems to outperform the resection margin status as a prognostic factor, and the recorded differences between R1 and R0 resections are equalized. Properly and accurately defining this patient group is a future challenge in the field of the surgical treatment of colorectal cancer liver metastases.

## 1. Introduction

Metastatic colorectal cancer is associated with a rather dismal 5-year overall survival of 14% [1]. Liver is the most commonly affected organ. Improved 5-year survival rates, even up to 58% after successful hepatic resections, for metastases confined to the liver, have been reported [2]. Therefore, surgery, in the form of hepatic resection with clear resection margins, can assign these stage IV colorectal cancer patients with resectable colorectal cancer liver metastases (CRLM) into a prognostic group with an associated overall survival pretty much similar to stage III colorectal cancer [3]. The technical definition of resectability has, notably, evolved over time with an obvious trend towards the expansion of the indications for surgery. In general, the disease is considered resectable as long as complete macroscopic resection is feasible, while maintaining at least a 30% future liver remnant or a remnant liver to body weight ratio >0.5 [4]. However, there are patients eligible for hepatectomy according to the technical criteria that the disease relapses shortly after the operation and ultimately do not experience benefits, in terms of survival, out of this approach. Indeed, as much as half of the patients submitted to hepatectomy for CRLM will develop widespread systemic disease within 3 years of resection [5].

The proper patient selection relies upon the identification of the risk factors behind the devastating event of early recurrence following hepatic resection for CRLM. In 1999, Fong et al. published a classic, in the field of liver surgery, analysis of 1001 patients submitted to hepatectomy for CRLM aiming to define the risk factors for recurrence after hepatic resection on the background of metastatic colorectal cancer [6]. The authors identified seven factors as independent predictors of poor long-term outcome: (1) Positive margin, (2) Extrahepatic disease, (3) Node-positive primary tumor, (4) Disease-free interval, from the diagnosis of the primary tumor to the development of the metastases, less than 12 months, (5) Number of hepatic tumors > 1, (6) Largest hepatic tumor larger than 5 cm in diameter, and (7) Carcinoembryonic antigen (CEA) levels > 200 ng/mL [6]. It becomes obvious that apart from the technical aspects of resectability, oncological criteria should be taken into account, as well, when deciding the treatment plan in patients with CRLM [7]. In practice, parameters such as the number of lesions, the possible presence of extrahepatic disease, and, in general, the criteria highlighted by Fong et al. [6], in their classic report, can predict a higher likelihood of longer disease-free survival [7]. Surgery is probably not the best option, at least upfront, when these risk factors are present.

These, not modifiable in their majority by treatment interventions, oncological criteria practically aim in calibrating and classifying the biology and the aggressiveness of the disease. However, a factor that could be modified, as it is decisively influenced by the surgical technique, is the resection margin width. Certainly, a hepatectomy that results in an incomplete tumor resection, in terms of leaving macroscopic residual tumor in the future liver remnant (R2 resection), is not associated with survival benefits. However, the prognostic implications of a microscopically positive surgical margin or a clear margin of less than 1 mm (R1) on pathology have been debatable. Although it has been a field of extensive research, the relevant literature often reports contradictory results. The purpose of the present study was to define, assess the risk factors for, and, ultimately, analyze the effect that an R1 hepatic resection for CRLM might have on local recurrence rates and long-term prognosis by reviewing the relevant literature.

## 2. Risk Factors for R1 Resection

An important aspect before assessing the prognostic correspondence of an R1 hepatic resection for CRLM is to identify patients at risk for such a resection. Welsh et al. developed a predictive index for quantifying the likelihood of an R1 hepatic resection for CRLM by studying a cohort of 929 patients. The authors reported an R1 resection incidence of 8.8%, while they identified five risk factors as independent predictors of an R1 resection: (1) Non-anatomical resection, (2) >3 hepatic metastases involving >50% of the liver parenchyma, (3) Bilobar disease, (4) Repeat hepatic resection, and (5) Abnormal preoperative liver function tests [8].

RAS mutations, which have been linked to more invasive and migratory tumor biology and a poor response to modern chemotherapeutic agents, have been implemented, as well. Brudvik et al. studied 663 patients with CRLM, of whom 229 had mutant RAS. In this study, the positive margin rate, defined as tumor cells < 1 mm from the resection margin, was 11.4% in the mutant RAS group and 5.4% in the wild-type RAS group (*p* = 0.007). The only factors associated with a positive margin were RAS mutation status (*p* = 0.005) and carcinoembryonic antigen levels of more than 4.5 ng/mL (*p* = 0.026). Furthermore, the authors analyzed the patients presenting with metachronous liver metastases and reported that those with mutant RAS had narrower clear resection margins during hepatectomy (median 4 mm vs. 7 mm—*p* = 0.031) [9].

The role of preoperative chemotherapy on resection margin status has been also investigated. Solaini et al. conducted a retrospective analysis, but with a propensity score matched analysis, and reported that preoperative chemotherapy was significantly associated with the rate of positive resection margin (25.5% vs. 8.5%) [10]. Aligned in the same direction, a multicenter study by Benedetti et al. analyzed a cohort of 3387 patients who underwent open or laparoscopic liver resection for CRLM in nine European high-volume referral centers. According to the results, the risk factors for R1 resection were: (1) The type of resection (non-anatomic or anatomic), (2) The number of nodules, and (3) The size of the tumor. In regard to the laparoscopic group specifically, blood loss proved to be a risk factor, whereas the Pringle maneuver had a protective effect against an R1 resection. The predictive size of a tumor for R1 resection was >45 mm for the open surgery and > 30 mm for the laparoscopic approach; the presence of more than two metastases increased the risk in both groups, while blood loss of more than 350 cc increased the risk in the laparoscopic approach [11].

It becomes clear that an R1 hepatic resection should not be considered solely synonymous to a poor surgical technique because the oncological characteristics of the metastatic disease could occasionally compromise the goal for an R0 resection. The presence of parameters that predict aggressive disease biology such as the increased number of lesions, the presence of bilobar disease, the mutant RAS status, and the poor response to chemotherapy could increase the likelihood for an R1 resection even when extensive hepatic resections, aiming to guarantee clear resection margins, are undertaken.

## 3. Definition and Prognostic Implications of R1 Resection

In general, curative surgery prerequisites the complete resection of the tumor with clear pathological resection margins, i.e., an R0 resection. Aligned with this dogma of surgical oncology, multiple studies in the early 2000s analyzing the results of hepatic resection on the background of CRLM have demonstrated that when a microscopically positive resection margin, i.e., an R1 resection, is the case, then a worse overall survival should be anticipated. More specifically, 5-year survival rates following a microscopically negative R0 resection and an R1 resection have been reported to range from 37% to 64%, and less than 20%, respectively [2,12,13,14]. However, de Haas et al., in their 2008 study with 436 patients treated with combined modality therapy utilizing chemotherapy and surgical resection, directly questioned these results. The authors reported that R1 resection had comparable long-term survival outcomes to R0 resection, i.e., 5-year overall survival rates were similar, i.e., 61% vs. 57%, between patients who had undergone an R0 or an R1 resection, respectively [15]. The study was greeted with skepticism by the surgical community and criticized for its methodology. However, it created the necessary creative doubt in regard to the actual impact of the resection margin status on overall survival. The logical assumption was that additional factors, besides the resection margin status, acting either synergically or independently could decisively influence the prognosis of patients with CRLM.

Traditionally, an R1 resection is considered a resection where, although there is no macroscopic tumor involvement on the resection margin, the pathology report highlights the microscopic resection margin as infiltrated by tumor cells. However, in 2015, the EGOSLIM (Expert Group on OncoSurgery management of Liver Metastases) group suggested that a minimal surgical clearance margin of 1 mm can be considered adequately safe when performing hepatic resection for CRLM [16]. This statement decisively influenced the definition of R1 resection as a resection with at least 1 mm of clear resection margin. However, in the literature, a microscopically positive margin (R1) has been commonly equated with either an involved margin (margin width = 0 mm) or a margin width of less than 1 mm. Unavoidably, the absence of a universally adopted definition acted as the starting point for problems with the interpretation of the results out of different studies. In practice, the proposal of the >1 mm clear margin definition of R0 resection appears justified as long as a notable difference in prognosis can be documented in the cohorts of patients created when the two definitions are separately applied.

Wang et al. aimed to test the validity of the definition of R1 resection focusing especially on patients with a margin width of 0–1 mm, a group of patients that have been inconsistently classified in either the R0 or R1 categories. The authors studied 633 patients who underwent a resection of CRLM and reported that a margin width of ≥1 mm was associated with improved survival vs. a sub-mm margin (65 vs. 36 months—*p* = 0.03) or an involved margin (65 vs. 33 months—*p* < 0.001) [17]. Using the same methodology, another study with 2368 patients submitted to a liver resection for CRLM at the Memorial Sloan Kettering Cancer Center compared survival according to the resection margin width. According to the results, the median overall survival of the R1 (0 mm), 0.1 to 0.9 mm, 1 to 9 mm, and 10 mm or more groups were 32, 40, 53, and 56 months, respectively. Compared with R1 resection (0 mm), all margin widths, including sub-mm margins, correlated with prolonged overall survival (*p* < 0.05). The authors concluded that wide margins should be the goal whenever possible, but resection should not be precluded if narrow margins are anticipated, as submillimeter margin clearance is also associated with improved survival [18].

The existing reports on the subject are mainly retrospective in nature with significant limitations. Propensity score matching is a statistical technique that has been widely used in order to reduce confounding biases such as selection bias in observational studies. A study by Sakai et al. represents an example on how propensity scores can alter the results of an observational study. The authors evaluated the influence of R1 resection on recurrence patterns and prognosis in 232 patients submitted to hepatic resection for CRLM. According to the results, patients with R1 resection had significantly poorer recurrence-free survival and overall survival compared to patients with R0 resection. However, after propensity score matching, there were no significant differences in recurrence-free survival and overall survival associated with the margin status. The authors concluded that among the group of patients with similar characteristics, as created by the propensity score matching, R1 resection does not seem to affect long-term outcomes and the R1 resection status should be regarded as an indicator of aggressive tumor biology [19].

In general, achieving an R0 resection with at least 1 mm of clear resection margin should always be the goal of a hepatic resection for CRLM because this seems to be consistently the safest way to achieve optimal long-term results. However, several technical and disease-related factors could lead to a suboptimal, i.e., less than 1 mm, resection margin. This R1 resection patient group exhibits notable heterogeneity in regard to prognosis. More specifically, among R1 resections, there are patients with a prognosis pretty much similar to R0 resections, while there are also patients with significantly worse prognosis compared to R0 resections. Within this context, adapting the definition of an R0 resection as a resection with at least 1 mm of clear resection margin appears justified. In regard to R1 resections, the actual challenge is to accurately define the oncological, technical, and clinical parameters that could neutralize the possible prognostic handicap associated with this certain resection margin status.

## 4. Factors That Could Neutralize the Effect of an R1 Resection

As the multimodality treatment of metastatic colorectal cancer is heading towards the most extensive end and a notable expansion of the indications for surgery, more and more patients with borderline resectable CRLM will become surgical candidates. An increased incidence of R1 resections should be anticipated out of this aggressive surgical strategy. The quest for factors or therapeutic interventions that could neutralize the possible prognostic handicap associated with an R1 resection appears justified. Factors such as the administration and the response to neoadjuvant chemotherapy, the KRAS mutational status, and other somatic mutations have been tested in this direction. A study that reported favorable results out of the administration of preoperative chemotherapy on the prognosis of R1 hepatic resections (microscopically infiltrated resection margin—0 mm) for CRLM was the study by Ayez et al. In this study, a total of 264 patients were divided into two groups with the criterion of neoadjuvant chemotherapy. According to the results, the median disease-free survival and overall survival of R1 patients that did not received chemotherapy was significantly worse than R0 patients (8 vs. 17 months—*p* < 0.001 and 30 vs. 53 months—*p* < 0.001, respectively), whereas this difference was not significant in patients after neoadjuvant chemotherapy (9 vs. 18 months—*p* = 0.303, and 65 months vs. result not reached—*p* = 0.645) [20].

However, there were also studies that did not confirm the efficacious effect of chemotherapy [21]. Pandanaboyana et al., for instance, analyzed 1255 patients that underwent resection for CLRM and reported a median overall survival and recurrence-free survival of 2.7 and 1.52 years, respectively, for the R0 group and 2.28 and 1.04 years, respectively, for the R1 group (*p* < 0.0001). The intrahepatic recurrence was higher in the R1 group (33.8 vs. 16.4%—*p* = 0.0001) and neoadjuvant chemotherapy did not seem to improve survival or have an impact on recurrence or reduce the need to redo liver surgery for recurrence in R1 patients [22]. Similarly, Ardito et al., in their study with 1428 resection areas in a total of 421 patients, reported that surgical margin recurrence after preoperative chemotherapy for CRLM was still significantly higher after R1 resection than it was after R0 resection and recommended R0 resections whenever technically achievable, as well as in patients treated by modern preoperative chemotherapy [23].

The factor “response to neoadjuvant chemotherapy” was evaluated by Laurent et al. who compared the oncologic outcomes after R0 and R1 (0 mm) resections in the era of modern chemotherapy in 191 patients [24]. The R1 resection rate (10%) was comparable in patients treated or not by preoperative chemotherapy. In general, the R1 status was associated with more intrahepatic recurrences and worse overall (44% vs. 61%—*p* = 0.047) and disease-free survival (8% vs. 26%—*p* = 0.082) compared to R0 resection. However, in the patient group that responded well to neoadjuvant chemotherapy, R1 resection, although it was associated with more intrahepatic recurrences, did not affect overall survival. On the other hand, it was postoperative chemotherapy that seemed to exert a protective, against recurrences, effect irrespective of the resection margin status [24].

The importance of RAS mutation status on the prognosis of metastatic colorectal cancer is becoming more and more appreciated. A study by Brudvik et al. highlighted RAS mutation as equally important, prognostic wise, as the primary tumor lymph node status and the diameter of the largest hepatic metastasis of more than 5 cm [25]. In a similar manner, Margonis et al. analyzed 485 CRLM patients with known KRAS mutational status. Approximately, two-thirds of tumors were KRAS wild-type (63.3%), while 36.7% had KRAS mutations. An R1 resection was associated with worse 5-year overall survival compared with R0 (42.4% vs. 57.1%—*p* = 0.001). After controlling for KRAS status, the survival benefit associated with an R0 resection persisted only among patients with wild-type KRAS tumors. In contrast, the surgical margin had no impact on overall survival among patients with mutant KRAS tumors (5-year overall survival: R0, 40.7% vs. R1, 46.7%—*p* = 0.348) [26]. On the other hand, Xu et al. analyzed 214 CRLM patients and evaluated the impact of these two parameters, concomitantly, on the prognostic significance of the resection margin status, i.e., the KRAS mutation status and the response to neoadjuvant chemotherapy. The authors showed that R1 resection impacted patients’ overall survival, i.e., 53.2% for R0 resections vs. 38.2% for R1 resections (*p* = 0.001). However, in the wild-type KRAS patients who responded to chemotherapy, R1 reached a similar overall survival to those who underwent R0 resection. For the RAS mutated and no response to chemotherapy subgroup, overall survival was significantly worse for those who underwent an R1 resection. The authors concluded that R1 resection margin is only acceptable in wild-type KRAS patients who respond to chemotherapy [27].

The possible selection bias in the previous two studies could explain the conflicting results. However, despite these differences, it becomes clear that factors that reflect the biology of the disease, such as the KRAS mutation status or the response to chemotherapy, appear as especially important determinants of prognosis. These factors could even possibly outperform the prognostic value of the resection margin status [26,27]. However, apart from the RAS mutation status, other somatic mutations have been also associated with the surgical margin status in regard to the incidence of local recurrence and the oncologic outcomes after hepatic resections for CRLM. In support of the above, Nishioka et al. highlighted the presence of extrahepatic disease, >8 cycles of preoperative chemotherapy, tumor viability ≥ 50% (HR, 1.55; *p* = 0.007), RAS/TP53 co-mutation (HR, 1.69; *p* = 0.001), and SMAD4 mutation (HR, 2.44; *p* < 0.001) but not surgical margin status to be independently associated with poor overall survival [28].

The ongoing research in the field has highlighted certain parameters as significant determinants of prognosis on the background of an R1 resection for CRLM. The impact of factors, such as the administration of and the response to neoadjuvant chemotherapy, the KRAS mutational status, and other somatic mutations, on the survival of patients is increasingly appreciated. The incorporation of these data on the treatment algorithm could more accurately define the patient group that would experience benefits, in terms of survival, out of an aggressive surgical strategy for patients with CRLM.

## 5. Discussion

The treatment of patients with CRLM requires the close collaboration of several involved specialties. Getting patients eligible for a hepatic resection provides the only chance for a favorable outcome. It becomes clear that this approach prerequisites an aggressive surgical management and, in many cases, the expansion of the indications for hepatectomy. However, without proper patient selection, the results are not always acceptable. The clinical scenario of an overwhelming disease relapse soon after the hepatectomy is not that rare. More than 20 years ago, Fong et al. set the scene for the comprehension of the behavior of metastatic liver disease from a colorectal primary cancer by identifying risk factors for recurrence after hepatectomy [6].

Nowadays, more factors are continuously being added to the equation. Margonis et al. aimed to develop a clinical risk score for resectable CRLM by combining clinicopathological and clinically available biological indicators, including KRAS, and, ultimately, update the classic Fong criteria [29]. The result was the GAME score, which was calculated by allocating points to each patient according to the presence of these predictive factors: KRAS-mutated tumors (1 point); carcinoembryonic antigen level 20 ng/mL or more (1 point); primary tumor lymph node metastasis (1 point); Tumor Burden Score between 3 and 8 (1 point) or 9 and over (2 points); and extrahepatic disease (2 points). A high-risk group, i.e., GAME score of at least 4 points, had a 5-year OS rate of 11% compared with 73.4% for the low-risk group (GAME score 0–1 point) [29].

In general, the issue of resection margin status and its prognostic implications is longstanding. Achieving an R0 resection is always the desired result, but in many cases, this cannot be achieved. The majority of literature reports have highlighted the detrimental effect of R1 resections on local recurrence and overall survival [12,13,14]. However, there are indeed studies that degraded the prognostic handicap as a consequence of an R1 resection in selected patients and highlighted the presence of RAS mutations, the response to chemotherapy, and, in general, the biology of the disease as important, if not the determinant, prognostic factors. Wang et al. evaluated the effects of surgical margins on oncologic outcomes with regard to the GAME prognostic risk score [29,30]. A cohort of 661 patients was divided into low- or medium-risk (GAME 0–3, 513 patients/124 R1 resections) and high-risk (GAME score 4–7, 147 patients/35 R1 resections) groups, and the effects of margin status on the overall survival and recurrence-free survival rate were examined. In general, R1 resection was associated with worse long-term results in the low- or medium-risk group, but in the high-risk group, no significant difference was found in median overall survival and recurrence-free survival among patients with R0 or R1 resection [30]. Therefore, margin clearance only improved survival rates in patients with a disease of good biology but had no effect on the long term in patients with more aggressive and difficult-to-control, with systemic therapy, disease.

A valid way to increase the pool of patients eligible for hepatic resection on the background of CRLM is to perform tissue preserving hepatectomies. However, in order to preserve more of the hepatic parenchyma, the preservation of crucial intrahepatic vessels appears necessary. This approach that often entails the marginal detachment of CRLM from these crucial intrahepatic vessels, usually with the use of intraoperative ultrasound imaging, does not fulfill the definition of an R0 resection [31]. The term R1 vascular resection has been proposed in order to distinguish these resections from the contemporary R1 parenchymal resections, as significant prognostic differences between these two R1 resection subgroups have been hypothesized [31]. Indeed, Vigano et al. reported that R1 vascular resections achieve outcomes equivalent to true R0 resections, while R1 parenchymal resections were associated with three times higher local recurrence rates compared to R0 resection [31]. The presence of a halo of proliferating tumor cells infiltrating the surrounding liver parenchyma around the metastasis could possibly explain the inferior results for the R1 parenchymal resections [32]. On the other hand, the vessels could act as an efficient boundary preventing tumor spread and explaining the favorable R1 vascular prognostic implications. However, factors that predict a disease of aggressive biology, such as the replacement histopathological growth pattern of CRLM or the presence of KAS mutations, could render marginal resection types such as the R1 vascular resection insufficient compared to true R0 resections [33,34,35,36].With all the constantly emerging and often conflicting literature data, an interesting aspect of the prognostic implications of an R1 resection is how this issue is dealt with by those who take part in the decision-making process of CRLM patients, i.e., the surgical community. An interesting study was a study by Vigano et al. who conducted a 19-question survey, completed by 276 hepatobiliary surgeons worldwide, regarding R1 resections for CRLM. According to this survey, 90% of the responders reported a negative impact of R1 resection (74% on local recurrence, 31% on hepatic recurrence, and 36% on survival), but 50% considered that an R1 resection is sometimes required in order to increase resectability. Nine out of ten responders suggested that the impact of R1 resection could be modulated by the response to chemotherapy and the characteristics of the CRLM. About 50% considered the risk of R1 resection to be an indication for preoperative chemotherapy in patients who otherwise underwent upfront resection, and 40% would modify the chemotherapy regimen when the tumor response was not optimal in order to guarantee an R0 resection. Nevertheless, 80% would schedule an R1 resection for multiple bilobar diseases that, however, responded sufficiently to chemotherapy [33]. An aggressive surgical approach that does consider an R1 resection as a technical failure but rather an expected outcome and part of the modern multidisciplinary management of CRLM can be elicited out of the responses.

## 6. Conclusions

In conclusion, achieving an R0 hepatic resection, optimally with more than 1 mm of clear margin, for CRLM should always be the goal. However, in the era of the multimodality treatment of liver metastatic colorectal cancer, an R1 resection might be the cost of increasing the pool of patients finally eligible for resection. In general, R1 resections are associated with local recurrence rates and long-term results worse than R0 resections but are still better than no resection. However, there are patients in whom the aggressive disease biology outperforms the resection margin status as a prognostic factor, and the recorded—in the whole CRLM patient cohort—differences between R1 and R0 resections are equalized. Properly defining this patient group is a future challenge in the field of the surgical treatment of CRLM.

## Data Availability

Not applicable.
